# Causal Inference Analysis for Poorly Soluble Low Toxicity Particles, Lung Function, and Malignancy

**DOI:** 10.3389/fpubh.2022.863402

**Published:** 2022-07-05

**Authors:** Philip Harber

**Affiliations:** Mel and Enid Zuckerman College of Public Health, University of Arizona, Tucson, AZ, United States

**Keywords:** causal inference analysis, directed acyclic graph, carbon black, chronic obstructive pulmonary disease (COPD), lung cancer, particulate toxicity, causation analysis, pulmonary inflammation

## Abstract

Poorly soluble low toxicity particles such as carbon black and titanium dioxide have raised concern about possible nonmalignant and malignant pulmonary effects. This paper illustrates application of causal inference analysis to assessing these effects. A framework for analysis is created using directed acyclic graphs to define pathways from exposure to potential lung cancer or chronic airflow obstruction outcomes. Directed acyclic graphs define influences of confounders, backdoor pathways, and analytic models. Potential mechanistic pathways such as intermediate pulmonary inflammation are illustrated. An overview of available data for each of the inter-node links is presented. Individual empirical epidemiologic studies have limited ability to confirm mechanisms of potential causal relationships due to the complexity of causal pathways and the extended time course over which disease may develop. Therefore, an explicit conceptual and graphical framework to facilitate synthesizing data from several studies to consider pulmonary inflammation as a common pathway for both chronic airflow obstruction and lung cancer is suggested. These methods are useful to clarify potential bona fide and artifactual observed relationships. They also delineate variables which should be included in analytic models for single study data and biologically relevant variables unlikely to be available from a single study.

## Introduction

Poorly soluble low toxicity particles (PSLTs) have received increased interest over the past few years. Concerns about possible malignant or nonmalignant pulmonary disease effects are being addressed. However, available epidemiologic data leave considerable uncertainty about the presence and magnitude of significant effects in humans. In general, studies of chronic pulmonary disorders (e.g., chronic airflow obstruction, CAO) and malignancies such as lung cancer (CA) are particularly challenging because of long latencies and major changes in typical workplace exposures over time. Although more mechanistic data are suggested by animal and *in vitro* studies, the applicability to human health is constrained by species differences and differences in toxokinetics.

### Causal Analysis

Causal inference analysis (CIA) provides an explicit framework for identifying and representing interacting factors of different types in complex multi-step causal pathways (1). It is particularly useful for delineating variable sets to be considered as covariates, defining exposure variables more clearly, and identifying potential biases leading to misleading conclusions. These methods also allow explicit consideration of mediators between exposure and disease outcomes.

Causal considerations have long been implicit in science; research increasingly seeks to provide explanations rather than simple descriptions. A causal relationship between A and B (A→ B) may conceptualized as either deterministic or probabilistic. In a deterministic relationship, A is sufficient to cause B. It is probabilistic if an intervention setting A to a fixed value changes the probabilistic distribution of B. Current assessments of the likelihood that PSLT's produce CA and/or CAO follow the latter inferential probabilistic paradigm.

Significant conceptual advances concerning causation occurred in the early 20th and early 21st. In the early 1900's, Popper conceptualized that an explanation may be considered scientific if it is possible to refute it by empirical observations. Similarly, Fisher, Pearson, and others helped create the basis for modern statistical hypothesis testing and statistical inference techniques.

Observational studies such as those assessing exposures and long-term health in humans must consider multiple factors. Such factors may be empirically measurable for inclusion as variables in statistical calculation models or be unmeasured but of potential interest. Such factors may increase or decrease the apparent relationship between the predictors and outcomes of interest; they may also prevent identification of bona fide causal relationships or lead to the appearance of artifactual relationships. Confounders in regression models are a such empirically measurable factors worthy of consideration. Even unmeasurable factors should be considered for interpreting the significance of calculated models. Occasionally, a measurable instrumental variable may be used to infer an important unmeasured factor.

Focus upon causation evaluation methodologies reignited in the early 21st century to deal with this potential complexity. Causal inference analysis helps identify the set of variables, whether measurable or unmeasured, that should be considered. Directed acyclic graphs (DAGs) are increasingly used to both facilitate ascertaining estimands and to force logical thinking and explicit representation of how they might relate. This paper illustrates how such DAGS may be applied to understanding several potential PSLTs effects.

Using pulmonary inflammatory response as an example, the paper then illustrates how DAGs may be applied to consideration of underlying biologic mechanisms in a single study. It then extends these principles to suggest how approaches analogous to DAGs may facilitate explicitly expressing potential underlying mechanisms even if information comes from separate studies. While mechanistic reasoning is already extensively used, the graphic approach facilitates explicit and logical representation.

### Directed Acyclic Graphs (DAGs)

DAGs are based upon underlying quantitative probabilistic models as described by Greenland et al. in 1999 (2). These methods may be applied for both facilitating specific calculations and for forcing explicit consideration of potential relationships (1).

A directed acyclic graph is composed of a series of nodes and links. The graph is built by including the set of all variables that affect the relationship between the predictor and outcome. Thus, for a complex topic such as PSLT effects, the graph may become complex. In this paper, the graphs are built sequentially beginning with very simple relationships and then adding more considerations. By including a full set of variables, the formal graph approach helps determine those variables that would not affect the primary relationship of interest. The DAG may include a series of alternate pathways.

In a DAG, causal relationships must be both directed and unidirectional. The direction of causation is indicated by a single arrow. That is, A→ B and B→ A cannot both be included. Causation generally is probabilistic rather than deterministic. In addition to causal links (represented by arrows), associations not implying causation may also be shown (e.g., by dashed lines without arrows).

The DAG is acyclic, meaning that even with a complex series of nodes and links, a variable may not have a directed path leading back to itself. Thus, A→ B and A→ C→ D→ B may be present but A→ C→ B→ D→ A is not acceptable.

### Paper Overview

This paper applies these methods to examine the specific questions: Do PSLTs such as carbon black (CB) cause chronic airflow obstruction (CAO) or lung cancer (CA), and if so, is this mediated by inflammation? The paper is illustrative and does not presuppose that such relationships exist. The approach is described in three sections: Frameworks (Section Framework for DAG Analysis) describes use of directed acyclic graphs (DAGs) to frame hypotheses and identify variables appropriate for analysis. DAGs consist of a series of nodes and links representing segments of potential causal pathways between the exposure and the possible outcomes. They also represent links to variables, both measured and unmeasured, that should be considered to avoid misleading results. Data for some variables identified as relevant may be challenging to acquire in a single study. Available data (Section Available Data) provides examples of data available for the many of the individual causal pathway link segments, although they may not be directly combined in a specific quantitative model. Implications (Section Implications of the Causal Mechanistic Analysis) suggests approaches for understanding disease development mechanisms by considering information from multiple studies. Potential future studies for CAO and CA are suggested. The approach emphasizes how existing data may be applied rather than providing a comprehensive summary of all relevant research data.

These considerations may contribute to understanding of PSLT effects in several ways: 1) Graphical methods such as DAGs make assumptions about causal pathways explicit. 2) It encourages specifically stating the set of variables deemed necessary and sufficient for understanding causal associations. 3) Limitations of data likely to be available from individual studies are described. 4) Examples illustrate how appropriate or inappropriate variable selection or adjustments may impact accuracy of measures of association. 5) Specific suggestions for qualitative inferences from distinct studies are shown.

## Framework for DAG Analysis

This section describes applications of DAGs for explicitly representing a framework for causal relationships and lays the foundation for assessing the role of empirical data in Section Available Data in specifying the relevant DAG. This section also illustrates methods to reduce the likelihood of misleading results. It also suggests potential intermediate steps along the path from exposure to disease.

### Fundamental Hypothesis

Potential pathways from PSLT exposure to final disease outcomes (CAO and CA) are represented as nodes and links. For simplicity, the nodes (PSLT, CAO, and CA) are shown as binary elements (yes, no). However, these methods may be extended to graded effects (e.g., CAO might be represented with a quantified physiologic airflow obstruction), or the strength of a causal link may be quantified. The figures include a series of DAGs showing progressively richer causal networks. Relationships between nodes are represented by a line with an arrow if the relationship is causal, by dashed lines without an arrow if the association is not causal, and by lighter dashed lines if it may be artifactual.

**Figure 1:**
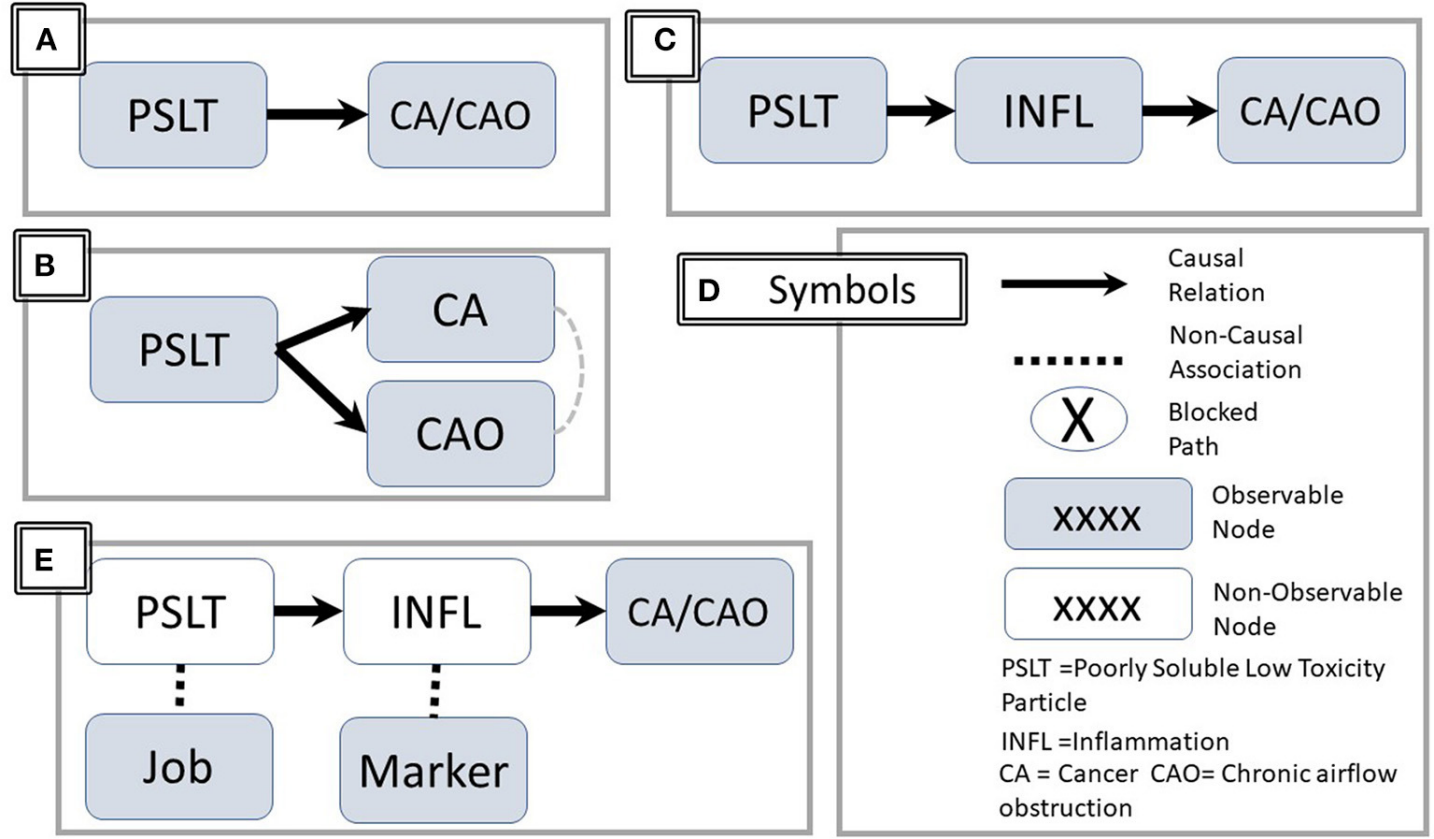
Directed Acyclic Graph (DAG) representations. The figure illustrates three causal sequences: **(A)** PSLT causes CA and CAO (solid line with arrowhead). **(B)** Clarification–the causation of CA and CAO are independent. However, a noncausal association may be observed between CA and CAO (dashed line without arrow). **(C)** PSLT acts through the mediator of inflammation. **(D)** Summary of representational symbols. **(E)** PSLT and INFL are indirectly assessed by associated job and biomarkers respectively. Unfilled nodes are not directly observable. PSLT, poorly soluble low toxicity particles; CA, cancer; CAO, chronic airflow obstruction; INFL, inflammation.

The traditional epidemiologic mortality study hypothesis asking whether PSLT predicts development of CA and CAO is shown in Figure 1A. This illustrates a direct causal or “front door path”. This is further clarified in Figure 1B, suggesting that PSLT exposure predicts CA and CAO, but they occur independently. In this circumstance, an association of CAO and CA would be observed even if there is no actual causal pathway between these outcomes. In addition, the apparent strength of association between PSLT and CA (e.g., odds ratio) may be attenuated by adjustment for the presence of CAO.

The mechanistic hypothesis that a causal relationship between PSLT and CAO or CA is mediated via pulmonary inflammation is shown in Figure 1C. For simplicity, the two health outcomes are combined in a single node. Figure 1D summarizes the common symbols.

However, there are several ambiguities in the apparently simple representation of the hypothesis.

- Even if inflammation is a critical intermediary step, inflammation *per se* may not be the final effector for producing airway damage or malignant cellular transformation (i.e., there may be additional intermediary steps).
- The number of potentially observable measures of inflammation, particularly in humans, is limited and may not truly reflect the *in vivo* inflammatory process.
- Inflammation is not a specific entity; rather, there are numerous inflammatory pathways, many of which are interactive and/or cross-regulatory. These are often assessed indirectly by measuring associated biomarkers (Figure 1E).
- “PSLT” is not a specific entity, and its definition is ambiguous. While many would agree that carbon black and titanium dioxide fall within this group, inclusion of other materials such as coal dust is less certain.
- Even for a specific agent, potential effects and pathways may depend upon the characteristics of size, particle size, dose, dose rate, charge, and surface properties. These were recently reviewed by Borm and Driscol (3).
- The exposure term (e.g., PSLT) may either refer to the specific agent or to a surrogate of exposure such as job title. Laboratory studies may use a chemically defined agent with specified dose or even assessed initial or retained dose. Neither exposure nor dose are typically precisely measured or controlled in human studies. Rather, these are assessed with proxy measures such as job title, job duration, etc. as approximate surrogate measures of actual dose (Figure 1E). The correlation between the surrogate and the actual dose is likely to be heterogeneous over time and among study sites.
- Time course: Inflammatory events early in the time course may initiate events ultimately leading to CAO/CA, but the mechanistic or exposure events later in the pathway may be different. Implications are discussed below.

**Figure 2:**
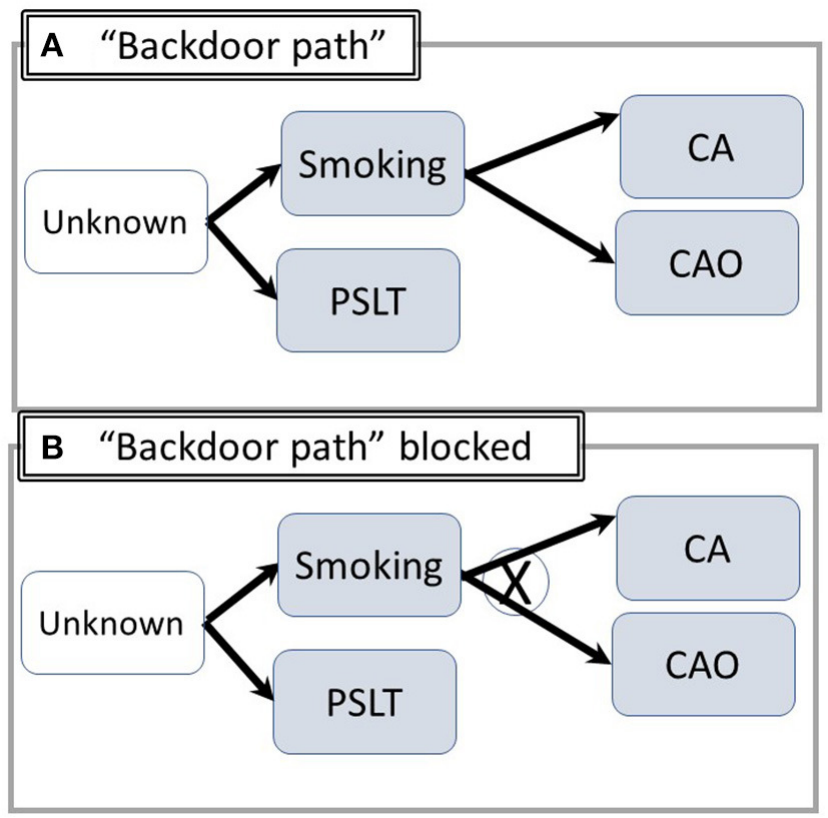
Utilizing DAG's to illustrate “Backdoor Paths” /confounding. **(A)** (“Backdoor Path”): Common unknown/unspecified factors increase the likelihood of both smoking and exposure. An artifactual apparent association between PSLT and CA/CAO may appear. X = pathway blocked by proper adjustment. **(B)** Adjustment for smoking blocks the pathway including PSLT-Unknown-CA (Abbreviations are summarized in Figure 1).

Explicit consideration of pathways involving both observable and significant but unknown (non-recorded) factors is essential for analysis and interpretation. Backdoor pathways, representing artifactual associations such as the well-known confounder of tobacco smoking are illustrated in Figure 2A. For example, smoking may be associated with exposure if common but unobserved socioeconomic or cultural factors increase the likelihood of working in a heavily exposed job and of smoking. Members of socially disadvantaged groups may be disproportionately likely to have jobs with heavy exposure and come from homes in polluted areas or have grown up with parental smoking. Adjustment for smoking, which produces both CA and CAO (Figure 2B) prevents the appearance of an incorrect causal association between PSLT and the outcomes.

**Figure 3:**
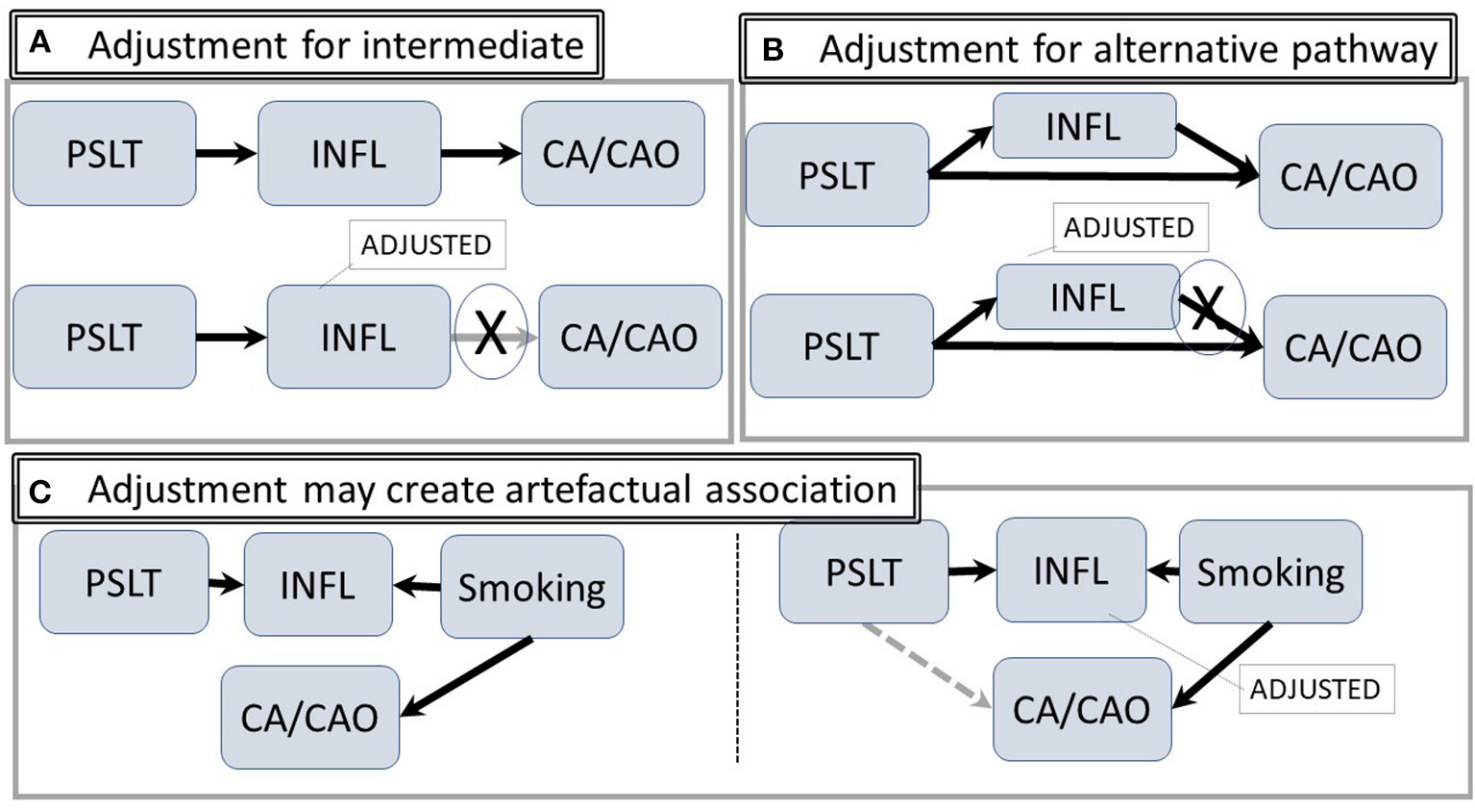
Intermediate and alternative pathways. **(A)** The hypothetical pathway from PSLT to CA/CAO is mediated through inflammation. Statistical adjustment for inflammation may attenuate or block the pathway. **(B)** Inflammation is an alternative pathway from exposure to CA/CAO; adjustment for inflammation will potentially modify the apparent strength of the association. **(C)** There is no path from PSLT to CA/CAO. However, adjusting for inflammation may open an artifactual pathway from PSLT to CA/CAO (shown in gray) (Abbreviations are the same as in Figure 1).

Conversely, adjustment for an intermediary step such as inflammation may reduce the likelihood of identifying a true relationship between PSLT and the outcomes. This potential overadjustment is illustrated in Figure 3A. Similar considerations apply if there are both a direct and an inflammation mediated pathway (Figure 3B) (The figure is simplified by assuming each of the nodes is directly observable). In contrast, adjustment for inflammation may create an artifactual association between PSLT and CA if both PSLT and smoking cause inflammation, but inflammation is not actually the mediator between PSLT and CA (see Figure 3C). In this “collider” situation, a false association between PSLT and CA would be observed if the PSLT-CA measure of association (e.g., odds ratio of CA on PSLT) is adjusted for inflammation (Adjustment for smoking would eliminate this effect, but it is preferable to simply not adjust for inflammation). Thus, assessment of which factors to use and when to adjust is facilitated by DAGs.

### Constraints Upon Observable Data in Human Studies

In addition to the consequences of long latency and occasional ambiguity in defining “exposure”, observational studies in humans require additional framework considerations. Such ambiguities may contribute to inconsistency among studies. These include distinguishing early vs. late steps in the causal chain and practical constraints upon measures of inflammation that may be collected noninvasively.

**Figure 4:**
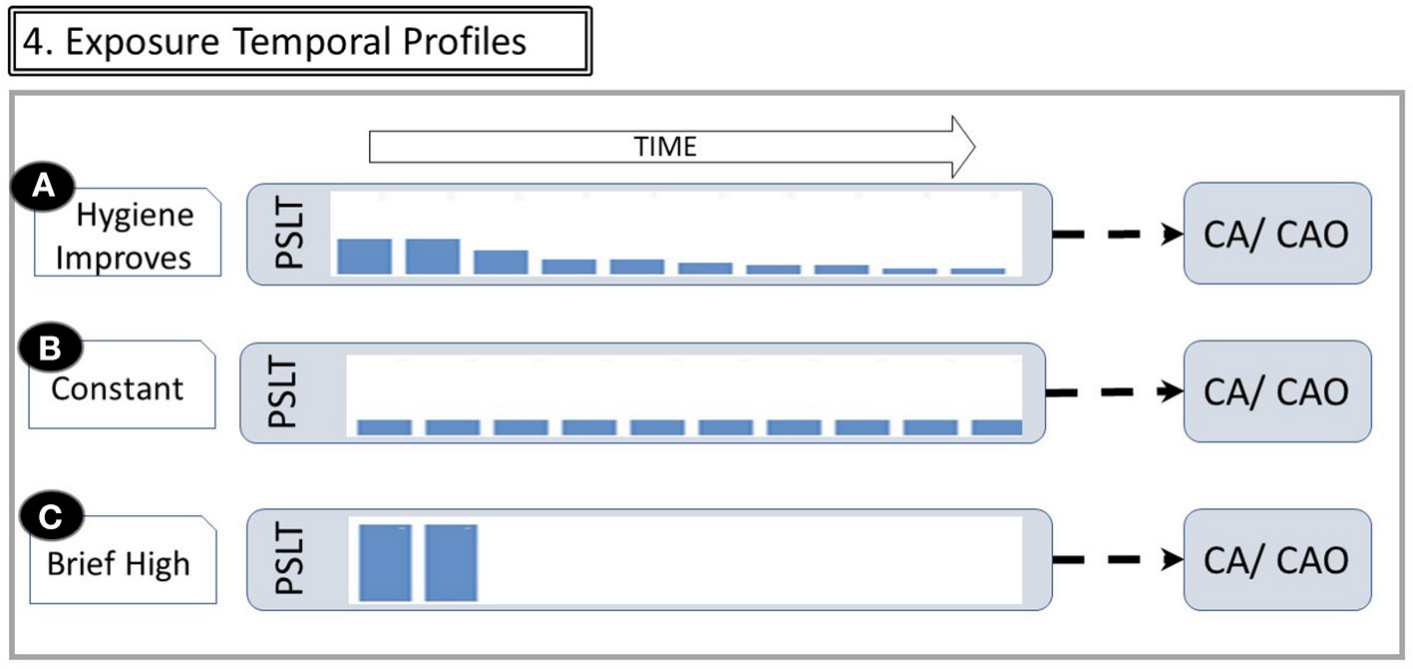
Exposure temporal profiles. The figure illustrates that the same cumulative dose may be received with different temporal patterns. **(A)** If exposure controls improve over time, long tenure workers have a relatively high exposure early in their career and then much less later. **(B)** Constant moderate exposure. **(C)** Extremely heavy exposure for short duration, then leaving industry with no subsequent exposure (Abbreviations are the same as in Figure 1). Changes in exposures over time are reflected by the height of bars within the PSLT node.

The importance of time is summarized in Figure 4. A study of whether PSLT predicts subsequent CAO or CA must cover a long time span. Even if the worker remains active in the industry until near the date at which diagnosis is made, it is necessary to reconstruct exposures many years in the past. This is particularly important since workplace air concentrations were often orders of magnitude greater in the past. In many instances, retrospective exposure assessment does not occur until near the date of diagnosis and is therefore subject to recall bias and/or missing data. Other workers may have left the industry long before recognition of CAO or CA, making cohort identification challenging.

Many studies utilize estimated cumulative exposure as the predictor of a possible health effect. However, the same cumulative exposure may be accrued with very different dose rates and latencies as shown in Figure 4. For example, some may have moderate exposure over many years, while others may have an early brief but very intense exposure. Misinterpretation of causal relationships may result from not considering the time varying nature of longitudinal exposures (4).

**Figure 5:**
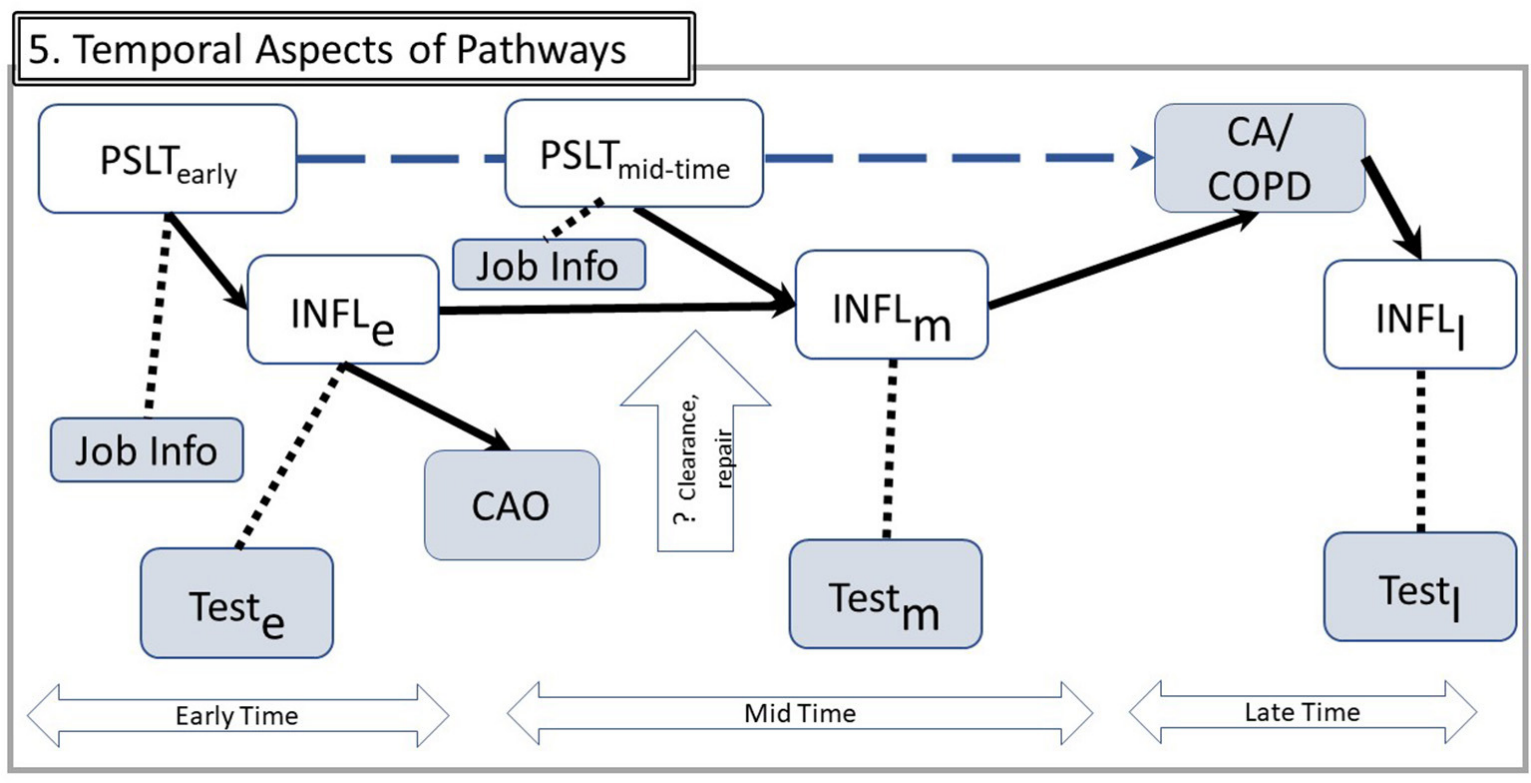
Temporal aspects of pathways. Using example from Figure 4A, the implications for observable data over time are illustrated. Time is divided in three eras: Early exposure, Mid for subsequent years of work, and Late after the disease outcome has been diagnosed. Inflammation processes in these three eras may involve different mechanisms, denoted by subscripts e, m, and l. Since INFL is not directly observable, it is assessed by relevant tests in each of these three times. The vertical open arrow indicates that some mechanisms such as particle clearance or repair mechanisms may be specific to one time era (e.g., some particle burden from early exposures may be cleared and therefore reduce transition from early to mid-inflammation). A hypothetical from PSLT to CA/CAO is shown by the dashed upper arrow. Other abbreviations are the same as in Figure 1.

Both biologic and operational consequences of the time course are illustrated in Figure 5, in which time is divided into early, mid, and late eras. The latter begins when the fully advanced disease is present.

Biologic considerations about PSLT's warrant consideration of the assumption that cumulative exposure is the optimal predictor. This is particularly true for PSLT's for which clearance and deposition may have thresholds, overloading, or significant nonlinearities. For example, clearance processes may be overwhelmed with very high early exposures but not with more moderate exposures. Limited epidemiologic adjustment methods such as overweighting or underweighting exposure by calendar date or excluding either recent or very remote exposures does not fully address biologic considerations such as short-term or long-term clearance, tissue repair, threshold doses, or other mechanistic considerations. Underestimation of remote exposures due to applying more recent data to earlier years (5) may lead to overestimation of the regression coefficient of CAO upon exposure.

The simple DAG of Figure 1 is augmented for analytic clarity in Figure 5. This framework expresses several possible considerations. Examples of several human inflammatory markers are shown in Table 1. Figure 5 represents that inflammation is better represented as a cascade of steps rather than as a single node, and there are multiple inflammatory pathways of which only some are likely to be relevant. Unlike animal and *in vitro* studies, the range of inflammatory biomarkers appropriate for human studies must be limited to noninvasive or minimally invasive procedures. Hence, the observable biomarker for each inflammatory step may be correlated with but not synonymous with the actual mediator (dashed line in the figure); the available biomarkers may therefore be indirect measures rather than direct assessment of the inflammatory steps. They are still useful, but they may only be loosely correlated to the actual inflammatory element.

**Table 1:** Markers of pulmonary inflammation (examples).

**Invasive:**
Animal sacrifice and histology/biomarker measurement
Bronchial biopsy, bronchial lavage
**Less invasive human sampling:**
Exhaled breath analysis (frozen or otherwise)
**Minimally invasive measures:**
Exhaled indicators: FeNO: (Exhaled nitric oxide)
(Subject to upper airway contamination)
(Short-term responsive)
(Does this reflect “important” mediator?)
**Non-pulmonary biomarkers:**
Nonspecific indicators e.g., CRP- C-reactive protein
CC16 – Club Cell 16: Mediator or a protector?
Micro-RNA
**Omics:**
Genomics
Epigenomics
Proteomics
Metabolomics
Targeted SNP's or hypothesis generating GWAS
**Markers of Effect**
Lung function
Quantitative CT scan

The figure also suggests that markers measured at various times may reflect various mechanistic aspects differing in their significance. Measurement of an early step may be more relevant for some outcomes in comparison to others. Measures of inflammation are more frequently assessed proximate to the health effect diagnosis (“mid”) than in the early years of exposure. Measurement of inflammatory markers in the several years immediately preceding diagnosis of CA or CAO may overlook the importance of inflammation in the earlier years in initiating the pathogenic sequence.

Reverse causation is another potential concern. For example, rather than PSLT leading to CAO and CA, it is possible that CAO increases the retained dose of PSLT if airway dysfunction reduces clearance of inhaled PSLT and therefore increases the effective retained dose of PSLT. It also is plausible that CA causes inflammation rather than the reverse causal relationship (in the ”late” era). This is illustrated by several of the empirical studies discussed below.

### Solution to Complexity

The complexity of a causal inference framework, as illustrated by the DAGs, does not imply it is impossibly challenging to understand whether there are bona fide causal relationships between PSLT and the health outcomes.

Much of the complexity is addressable by collecting complete and accurate data on as many variables as feasible in a specific study. This may require long-term studies of a worker group or acquiring data previously obtained (an example is provided in Section Implications of the Causal Mechanistic Analysis). Appropriate adjustments to block backdoor pathways, avoiding adjustments that may introduce artifactual biases, or use of analytical models incorporating do-calculus may help (6). Careful analysis may also identify variables that need not be studied, thereby improving study efficiency (7). In special well-circumscribed circumstances, estimation of effect in one population may be aided by incorporating data from other studies; Pearl applied terms such as transportability and meta-synthesis to these approaches (6, 7). While this paper emphasizes specifying the DAGs using expert knowledge derived from existing studies, causal structures may sometimes be derived from data themselves with causal distributional notation beyond associational measures (7).

Similarly, information necessary to thoroughly assess the postulated intermediary steps from a single work history—health outcome study. Rather, explicitly delineating the individual node and link segments permits considering available data for each of these segments. For some, analogous human data are available, and for others relevant animal data are applicable. Thus, explicit framework analysis promises to overcome inherent constraints of using a single study and integrating empirical data with conceptual understanding. This approach is illustrated in Part 2 below.

## Available Data

The complex causal inference links and nodes shown in Figure 5 make it unlikely that a single set of human data can traverse all the nodes in the causal pathway. However, data applicable to the each of the links in aggregate may guide consideration of mechanistic hypotheses. The analytic framework described in this paper illustrates how information of several types may be synthesized. It does not provide a specific method for calculating a precise quantitative measure of association (2).

The section includes examples of data types relevant to each of the segments linking particles to inflammation and inflammation to disease. Categories of supportive information include biologic plausibility, disease associations, common exposure associations, common genetic risk factors (e.g., proinflammatory genes), early/pre-lung cancer studies, common mediators, prospective population-based studies, consistency with mediators measured in animal toxicology studies, and internal consistency of postulated mechanisms.

### Overall Associations

Several epidemiologic studies have examined the hypotheses that PSLT produces CAO and that PSLT produces CA with studies of carbon black (CB). The CB-CAO relationship was addressed by studies of Harber in North America (8, 9) and Gardiner in Europe and the United Kingdom (5) examining the relationship between estimated cumulative exposure and respiratory outcomes. Both observed that chronic exposure led to a reduction of the forced expiratory volume in one second (FEV1), with the average slope of 0.7 ml and 1.2 m/ per cumulative mg-year/m^3^ inhalable dust respectively. And effect upon the forced vital capacity was observed in the North American but not the UK/European study. These were cross-sectional studies in which cumulative exposure was retrospectively estimated for the total duration of work for each subject; North American subjects had an average of 14.1 years of exposure (8, 9).

These studies illustrate the practical impact of retrospective exposure assessments. Cumulative exposure estimates were considerably higher in the Harber than the Gardiner studies since the former used all available data to assign exposures in the earlier calendar periods, whereas the latter applied measurements from the early 1990s to earlier years (5, 8). This methodologic difference is likely to account for differences in regression coefficients for FEV1. Neither considers the temporal pattern or dose rate.

Several epidemiologic mortality studies have addressed the relationship between CB and CA (10–13). In addition, several comprehensive reviews consider this possible association and pointed out the inconclusive nature of the studies (14).

### Indicators of Inflammation

As discussed in Section Framework for DAG Analysis, “inflammation” is itself complex since it changes over time and includes many alternate pathways. For human data to be germane to the mechanistic hypothesis under consideration, the measures must be minimally or noninvasive, capable of repeated measurement over time, and reasonably anticipated to correlate well with the actual biologic processes. Potential markers are shown in Table 1 and their timing is described in Figure 5.

### Associations Between Diseases

Cross-sectional studies show that several established lung diseases with persistent inflammation are associated with an increased cancer incidence (e.g., tuberculosis, idiopathic pulmonary fibrosis, and CAO) (15–17). These relationships might arise either because inflammation is an underlying cause of both CA and the nonmalignant disease or is a consequence rather than cause of the nonmalignant disease and/or CA. A recent review summarized data supporting a common antecedent for both CAO and CA (18). Many such studies suffer from only looking at “late stage” findings and depend upon fully established clinical diagnoses.

Longitudinal studies overcome this limitation. The US National Health and Nutrition Examination Survey (NHANES) identified 113 incident lung cancers during the 17 year follow-up (19). Moderate or severe CAO at baseline was associated with a significantly elevated risk of subsequent incident CA (OR = 2.6, CI = 1.5–3.8). Analogous data were reported by O'Callaghan (20). Thus, despite limitations, disease association studies provide some support for the proposed mechanism.

### Inflammation Concurrent With CA or CAO

Studies have also demonstrated that inflammation is frequently present when CAO or CA is diagnosed. Limited weight should be given to studies showing of inflammation at the time of CA diagnosis such as, the adverse prognostic impact of inflammation at the time of diagnosis (21, 22). These may be important for guiding treatment but have limited import for describing the significant pathway (see Figure 5).

### Associations Between Particle Exposure and Inflammation

Human and animal data support a role of pulmonary inflammation. These studies include both population-based air pollution studies with long-term exposures as well as shorter-term acute exposures (e.g., air pollution episodes, wildland fires), and laboratory studies in chambers. Animal studies have been particularly fruitful in showing that with adequate dose, PSLT's can lead to inflammation. This is likely to depend upon the dose time profile and may proceed through several distinct pathways. Human studies are more limited. For example, a study among CB workers and nonexposed controls found increased eosinophils with CB exposure. The differences in peripheral inflammatory markers between CB exposed and unexposed workers were more evident when analyses were stratified by smoking status (23, 24).

### Associations Between Inflammation and CA/CAO

Several prospective studies support causal associations between inflammation and CA and CAO. For example, in an 8 year follow-up, elevated C-reactive protein (CRP) was associated with an elevated risk of subsequent lung cancer (hazard ratio = 3.39) (25). Since the increased CRP antedated the CA, reverse causation (CA→ inflammation) is unlikely. An analogous study using high-sensitivity CRP was reported by Muller (26). Association of CRP with lung functional morbidity such as airway hyperresponsiveness or hospitalization has also been reported (27, 28). Genetic studies support this thesis (29, 30).

## Implications of the Causal Mechanistic Analysis

The two preceding sections establish an analytic framework and provide suggestive data applicable to each segment of the pathway. This part suggests their practical implications.

First, causal inferential mechanistic analysis is useful for identifying relevant factors to be considered and suggesting considerations for appropriate and inappropriate adjustments in specific statistical models. Additionally, analogous principles extend to synthesizing data across studies for inferential purposes and informing underlying mechanistic considerations.

Second, translating observed data into practical policy may benefit from mechanistic considerations. Dose and dose rate are particularly germane if the mechanism suggests that clearance may either reduce the effective dose or become saturated at high dose rates. Reliance upon estimated total cumulative dose is not robust to such considerations. In addition, it is useful to consider whether intermediary steps are potentially irreversible or may be counterbalanced by repair mechanisms. Irreversible mutational changes may be contrasted with inflammatory sequences subject to internal homeostatic controls. The analysis presented in this paper does not in itself address these questions specifically for the PSLT's, but it establishes a framework through which other data such as animal toxicology studies may be integrated with observable human data.

Third, these approaches facilitate planning future research studies to strengthen the empirical database. As shown in Section Framework for DAG Analysis, it is unlikely to be practical to measure inflammatory markers throughout a working lifetime and thereafter to link early inflammatory responses to subsequent CAO or CA identified decades later. However, the individual segments (node-link-node) may be studied, albeit in different individuals. For example, the relationship between PSLT and inflammation may be assessed in exposed workers with different temporal dose patterns (see Figure 3). Such results may contribute to assessing the significance of current lower-level industrial worker exposures and/or effects upon end-users. Similarly, resolution of inflammatory markers after cessation of exposure may be assessed.

**Figure 6:**
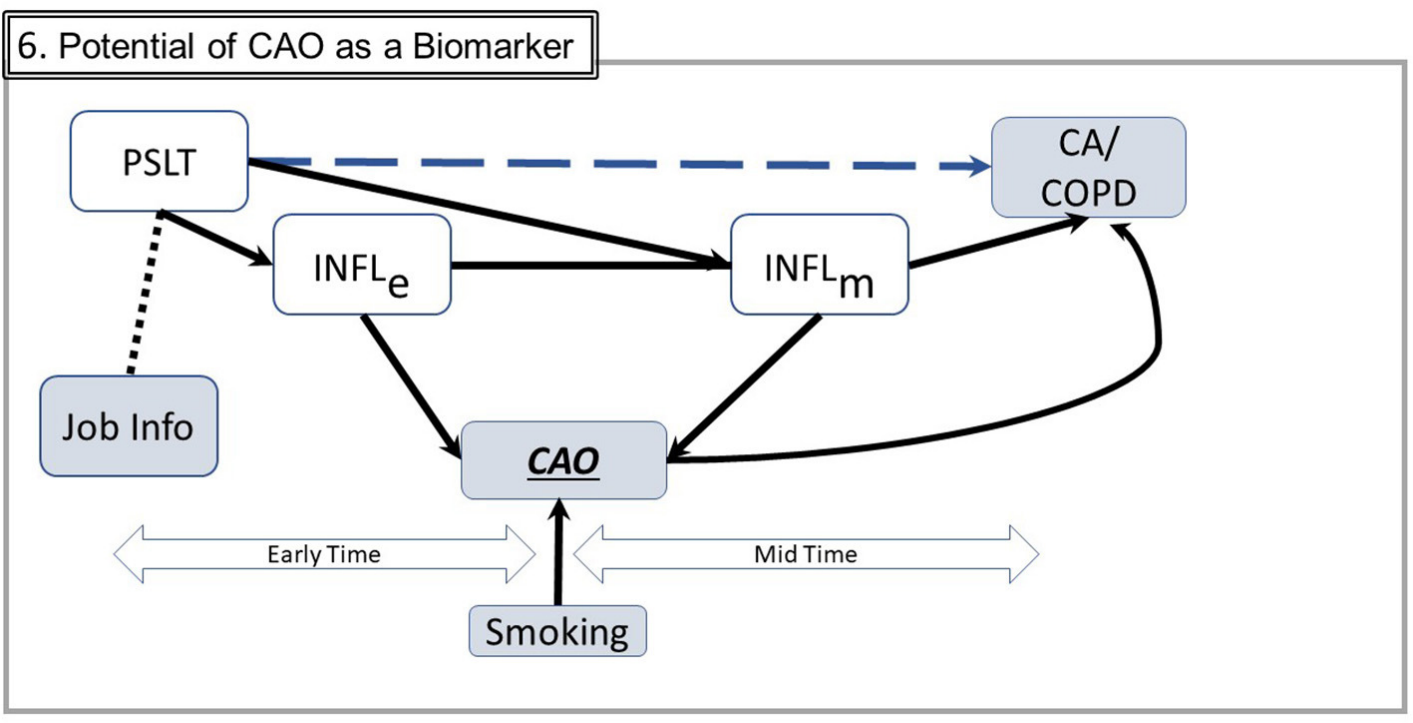
Potential of CAO as a biomarker. This figure includes simplified elements from Figure 5. CAO occurs both in the early and the mid times and therefore may be empirically ascertained throughout the long time from exposure to potential illness. In this figure, COPD is chronic obstructive pulmonary disease, a defined disease entity rather than a measurable trait such as CAO. Smoking, commonly the most important confounder, may generally be ascertained and subject to adjustment (Abbreviations are the same as in earlier figures; Several components from prior figures are not shown to facilitate graphical clarity).

Fourth, the different time courses for detecting CAO and CA might be leveraged to gain insight. Unlike CA, which is typically diagnosed at a specific “late” point in time, CAO develops gradually and is detectable early in its course well before advanced disease such as chronic obstructive pulmonary disease is present (see Figure 6). If inflammation is central to the development of both outcomes, monitoring CAO may provide insight into the temporal/concentration exposure characteristics associated with inflammatory responses. CAO is easily measured repetitively and noninvasively at low cost with spirometry or associated inflammation biomarkers such as exhaled nitric oxide. Assessing temporal course of CAO with different temporal exposure patterns would provide insight relevant to the significance of dose-time exposure patterns. Ongoing industry medical surveillance programs may already have data permitting such analyses. This approach, while currently hypothetical, may be practically implementable.

## Summary

Causal inference analysis such as use of DAGs is a very useful tool to help clarify many of the significant questions concerning the health significance of poorly soluble low toxicity particles. It is useful both for representing the set of appropriate variables and guiding analytic models for individual studies. Its principles of identifying relevant variables and their potential effects may be applied as a heuristic to graphically represent relationships explicitly to foster qualitative synthesis of information from disparate studies.

## Data Availability Statement

The original contributions presented in the study are included in the article/supplementary materials, further inquiries can be directed to the corresponding author.

## Author Contributions

The author confirms being the sole contributor of this work and has approved it for publication.

## Conflict of Interest

The author declares that the research was conducted in the absence of any commercial or financial relationships that could be construed as a potential conflict of interest.

## Publisher's Note

All claims expressed in this article are solely those of the authors and do not necessarily represent those of their affiliated organizations, or those of the publisher, the editors and the reviewers. Any product that may be evaluated in this article, or claim that may be made by its manufacturer, is not guaranteed or endorsed by the publisher.
